# The use of multisensory environments in children and adults with autism spectrum disorder: A systematic review

**DOI:** 10.1177/13623613251320424

**Published:** 2025-03-14

**Authors:** Simona Leonardi, Marcella Di Cara, Silvia Giliberto, Adriana Piccolo, Carmela De Domenico, Giulia Leonardi, Angelo Alito, Rosamaria Siracusano, Rocco Salvatore Calabrò, Angelo Quartarone, Francesca Cucinotta

**Affiliations:** 1 IRCCS Centro Neurolesi Bonino Pulejo, Messina, Italy; 2 Department of physical and rehabilitation medicine, University Hospital “G. Martino,” Messina, Italy; 3 Department of Biomedical, Dental Sciences and Morhological and Functional Images, University Hospital “G. Martino,” Messina, Italy; 4 Division of Child Neurology and Psychiatry, University Hospital Federico II, Napoli, Italy

**Keywords:** autism, autism spectrum disorders, intervention, multisensory environment, multisensory room, rehabilitation, sensory rooms, Snoezelen^®^, systematic review

## Abstract

Multisensory environment is a setting designed with activities and tools that offered sensory stimulation. Despite their widespread use, no evidence-based guidelines are currently available. The aim of this systematic review was to assess the impact of multisensory environment interventions in autism and to provide guidelines. We included all studies of multisensory environment interventions for autistic individuals retrieved from PubMed, Web of Science, and Science Direct up to 30 September 2024. Two researchers appraised the included literature and extracted the data. A total of 1247 unique records were screened for eligibility, and 10 studies were included. Data extraction included demographic characteristics, type of intervention, target symptoms, and outcome measures. Quality assessment tools included the Newcastle–Ottawa Scale and the Cochrane Risk of Bias for randomized controlled trials. The studies were synthesized narratively based on target symptoms. Four studies reported reductions in stereotypic behavior frequency in children and adults; while other studies suggest positive effects on sustained attention, and aggressive and sensory behaviors. Overall, there was insufficient evidence due to the paucity of literature, the significant variation between interventions, and the small sample sizes. Future research should aim to develop a structured intervention approach to address the common limitations of the included designs.

**Lay abstract:**

Multisensory rooms, also known as multisensory environments, are widely used in clinical practice and schools for autistic people. Despite their widespread use, their usefulness or effectiveness in achieving specific improvements is still unclear. We carry out a comprehensive and systematic quality assessment of all available studies to test the effectiveness of multisensory environment interventions in autism spectrum disorder and to explore what type of targeted intervention is needed to improve both core symptoms and associated features. The results show that multisensory environment could be a useful tool to modulate aggressive and stereotyped behaviors in autistic individuals. Although there is insufficient evidence to conclude on the efficacy of multisensory environment for other types of targets, overall, the results may provide valuable insights for the development of future studies concerning the utility of multisensory environment in therapeutic intervention.

## Introduction

A multisensory environment (MSE) is a structured space that provides stimuli through sophisticated equipment (Lancioni et al., 2002). This specialized environment is characterized by devices that provide a variety of visual, auditory, olfactory, tactile, vestibular, and proprioceptive sensory inputs (Breslin et al., 2020) through different modalities. This particular setting is inspired by an approach developed in 1970 by two therapists, Hulsegge and Verheul (2005). The Snoezelen method, a word derived from the Dutch words “snoffelen” (to explore) and “doezelen” (to relax), is based on the free exploration of the environment by the user. This intervention is characterized by extensive stimulation and is based on non-directive and non-threatening approaches, implemented in a playful context and aimed at achieving goals calibrated to the user’s needs, involving the psychomotor, cognitive, sensory-perceptual, affective-relational, and communicative domains (Hulsegge & Verheul, 2005).

While all Snoezelen rooms are considered a type of MSE, not all MSEs adhere to the specific Snoezelen approach (Eijgendaal et al., 2010). Snoezelen rooms are designed with particular equipment, such as adjustable lighting, soothing sounds, and tactile elements, all tailored to create a calming and exploratory experience (Hulsegge & Verheul, 2005). Conversely, other MSEs may incorporate a variety of sensory stimuli and therapeutic objectives that do not strictly align with the principles of the Snoezelen method (Lancioni et al., 2002). For example, some MSEs might focus on structured activities or specific therapeutic goals rather than fostering free exploration (Breslin et al., 2020). Therefore, while Snoezelen rooms exemplify a specific philosophy of sensory engagement, the broader category of MSEs encompasses a wider range of methodologies and purposes, each designed to meet diverse sensory needs and therapeutic goals.

In recent years, architect Lehman (2016) introduced the term sensory adaptive environments (SAEs) to refer to the ability of shared living environments to be adapted to the sensory needs of occupants through innovative technologies and interdisciplinary principles. The initial goal was to improve the well-being of people within built spaces; in particular, healthcare and educational environments have been the most explored in this field, demonstrating the importance of sensory experiences to engage and enjoy various settings (Williams et al., 2024).

Although sensory processing deficits have been noted since the first descriptions of autism spectrum disorders (ASDs; Ben-Sasson et al., 2009; Kanner, 1943), it was not until the Diagnostic and Statistical Manual of Mental Disorders, Fifth Edition (*DSM-*5; American Psychiatric Association, 2013) that sensory problems were included as a core symptom and diagnostic criterion. Individuals with ASD often experience challenges in sensory processing or invest particular interest in sensory aspects of the environment (Riboldi et al., 2024). These sensory abnormalities are estimated to be present in more than 90% of autistic individuals (Leekam et al., 2007).

According to *DSM*-5, sensory processing impairment includes hyper- or hypo-reactivity to unusual sensory stimuli or sensory interests in the environment. Hyperreactivity, characterized by an exaggerated response to sensory stimuli, appears to be the most prominent difference compared to neurotypical peers or other clinical groups (Baranek et al., 2006; Ben-Sasson et al., 2019). In contrast, hyporesponsiveness, characterized by diminished or absent responses to sensory stimuli, has also been demonstrated in other groups with clinical conditions (Ben-Sasson et al., 2019). In addition to high frequency, there also appears to be a broad impairment of multiple sensory domains (Leekam et al., 2007). Notably, all of these sensory abnormalities may co-occur and reproduce individually mixed clusters that need to be assessed and managed (Ben-Sasson et al., 2008), as they appear to be pervasive and persistent across all ages, affecting the development and adaptive behaviors of autistic children and adults (Leekam et al., 2007).

Given the sensory differences associated with ASD, the possibility to calibrate the sensory stimulation provided by MSE makes the environment predictable; this is a preferred condition for autistic people (Ashburner et al., 2013; Robertson & David, 2015) and could reduce feelings of anxiety (Boulter et al., 2014). MSEs can offer benefits that extend beyond therapeutic goals by fostering an immediate sense of comfort, reducing anxiety, and promoting self-regulation through sensory immersion (Baillon et al., 2002; Boulter et al., 2014). Specifically, MSEs allow for a controlled environment where stimuli can be tailored to the individual’s sensory profile, providing the flexibility to modulate hyper- or hypo-sensitivity across various sensory domains (Ashburner et al., 2013). These features permit a comprehensive response to the great heterogeneity of the autistic population, both in terms of sensory profiles and needs, and can offer a personalized intervention approach.

Moreover, these remarkable conditions, also applied to therapeutic intervention settings, could provide an opportunity to promote learning and meet their sensory needs (Baillon et al., 2002). Practitioner reports suggest that the use of MSE facilitates benefits for autistic children (Unwin et al., 2021), but despite its widespread use, there are no established guidelines to support the use of MSE with autistic individuals, and few studies have reported experiences autistic people.

In the last decade, MSEs have been increasingly modernized as technology has advanced, leading to the implementation of different models. Furthermore, their use, albeit with different methodologies and objectives, appears to be generally beneficial in several conditions, such as intellectual disability (Lotan & Gold, 2009), dementia (Chung et al., 1996), Alzheimer’s disease (Smith & D’Amico, 2020), and ASD. To date, MSEs are widely used in special schools for autistic children and are recommended as part of best practice in education (Altenmüller-Lewis, 2017; Department of Education, 2015).

To our knowledge, no systematic review has comprehensively evaluated the effectiveness of MSE interventions for ASD. The aim of this systematic review is to collect and synthesize literature evidence to assess the applicability and effectiveness of MSE for autistic people, both children and adults.

## Methods

### Information source and search strategy

This systematic review was registered in the PROSPERO database (CRD42024466984). It was conducted according to the Preferred Reporting Items for Systematic Reviews and Meta-Analyses (PRISMA-S; Page et al., 2021; Rethlefsen et al., 2021).

Articles were selected from three electronic databases: Science Direct (Elsevier), Web of Science, and PubMed (National Library of Medicine) up to 30 September 2024. The search included multiple terms related to “autism” OR “autisms” OR “autistic spectrum disorder” OR “autistic disorder” OR “ASD” AND “sensorial” OR “sensorially” OR “sensory” OR “sensorial” OR “sensorially” OR “sensory” OR “multisensorial” OR “multisensory” OR “snoezelen” AND “environ” OR “environment” OR “environments” OR “environment’s” OR “room,” which were listed in the Supplementary Material. In addition, the reference lists of included studies or reviews were searched to ensure that a comprehensive list of relevant articles was considered for inclusion.

### Eligibility criteria

We included articles that met the following criteria: (a) published in peer-reviewed journals; (b) randomized controlled trials (RCTs), both parallel or crossover studies, and uncontrolled clinical trials; despite the potential for bias, we decided to include case series studies (sample ⩾ 3) due to the paucity of literature; (c) an MSE used for therapeutic intervention; (d) individuals with an ASD diagnosis according to the Diagnostic and Statistical Manual of Mental Disorders (4th and 5th ed.; American Psychiatric Association, 2013) or ICD-9 or ICD-10 (DiSantostefano, 2009; Slee, 1978) of any age; (e) articles published in any language.

Exclusion criteria were (a) clinical case reports (sample < 3), letters to the editor, comments, conference abstracts, and reviews; (b) samples without a well-defined diagnosis of ASD; (c) reports with overlapping samples; where overlap was found, the largest study was included.

### Selection procedures

Study selection was conducted by two blinded authors (S.G., S.L.); in case of disagreement, a third author (M.D.C.) was involved who discussed the issue with the other authors and reached a consensus. If there was any doubt about inclusion, the article moves on to the next stage. Initially, titles and abstracts were screened, excluding those not pertinent. After the screening stage, full-text articles were assessed for eligibility, by two authors (SG and SL), independently, with 93.6% concordance. Discrepancies were resolved by the third author (M.D.C.).

### Data extraction and evaluation

To depict and assess study characteristics, all variables were coded as categorical and well-defined by one author (F.C.). The coding process included both intermediate and final agreement checks to ensure consistency and reliability. After the initial coding, two authors (S.L. and S.G.) independently reviewed and conformed to these metrics. Any discrepancies identified during intermediate checks were discussed and resolved through consensus among the authors, ensuring alignment before proceeding. At the final stage, a thorough review confirmed that full agreement had been achieved on the coding and synthesis of the articles. The data extraction processes included: (1) general information comprising first author, year of publication, country, and study design; (2) sample characteristics including sample size, gender distribution, mean, standard deviation and range of age, clinical diagnostic assessment performed, comorbid neuropsychiatric condition, the reported intellectual quotient and ASD severity, numerosity of each experimental group (when applicable); (3) intervention characteristics including aims and main effects, type and professional figure who carried out the intervention, number, duration and frequency of sessions, information regarding the setting of experimental intervention, and outcome measures.

### Strategy for data synthesis

It was not possible to calculate an average effect size because of the high heterogeneity in outcomes and outcome measures, and because 3 of the 10 studies used a case report design. Further subanalyses were performed according to (1) sample characteristics; (2) intervention type; (3) outcome measures; (4) synthesis of main effects.

### Quality assessment

The Newcastle–Ottawa Scale (NOS) tools were used for the non-randomized trials and the Cochrane Risk of Bias for RCT tools were used for the RCTs included in the review. The NOS assesses three broad domains: selection, comparability, and outcome. Selection assesses how well the study identifies and recruits its participants, rating higher those studies with clear inclusion and exclusion criteria or that used a well-defined population-based cohort, and lower those with convenience samples or unclear recruitment procedures. Comparability evaluates whether the studies controlled for confounding variables. For example, studies that adjusted for factors such as age, sex, or baseline health status received higher scores, while those that did not adjust for these factors received lower scores. Outcome or exposure measures the rigor of outcome procedures (e.g. well-validated outcome measure) and follow-up. Each domain is further subdivided into sub-domains and eight areas of bias, each of which receives a maximum of one star, except for comparability which receives a maximum of two stars. Specifically, bias due to (1) representativeness of the exposed cohort, (2) ascertainment of exposure, (3) selection of unexposed cohorts, (4) demonstration that the outcome of interest was not present at baseline, (5) comparisons between groups based on design or analysis, (6) outcome assessment, (7) sufficient follow-up time, (8) adequacy of follow-up of cohorts. Studies are rated from 0 to 9, with 0–3 (low quality), 4–6 (fair quality), and 7–9 (good/high quality; Wells et al., 2000). The Cochrane Risk of Bias Tool assesses internal validity by systematically evaluating seven key areas. These include randomization sequence, generation allocation, concealment, selective reporting, blinding of participants and personnel, blinding of outcome assessment, incomplete data results, and other biases. Random sequence generation analyses the quality of randomization and its freedom from manipulation or predictability, thereby reducing selection bias. The allocation concealment domain refers to the process of concealing the randomization sequence from both participants and investigators until assignments are made. The selective reporting domain assesses whether the study selectively reports outcomes based on the results. The blinding of participants and personnel domain assesses whether participants and study personnel were blinded to the treatment assignments, preventing participants and researchers from being influenced by their expectations of the effects of the intervention. The Blinding of Outcome Assessment domain assesses whether those responsible for measuring or assessing outcomes are blinded to treatment allocation; this is particularly important for subjective outcomes (e.g. pain) that may be influenced by prior knowledge of the treatment. The Incomplete Outcome Data domain assesses how the trial handles missing data from participants who drop out or are lost to follow-up to avoid bias in the results. The Other Bias domain covers biases that do not fit neatly into the other categories but can still affect the internal validity of the trial. It includes biases from issues such as attrition (stopping a trial early because of dramatic results), baseline imbalances (differences between groups at the start of the trial), and funding bias (the influence of funding sources on the study design or results; Higgins et al., 2011). The results were then converted to Agency for Healthcare Research and Quality (AHRQ) standards, which ultimately classify RCTs as “good quality,” “fair quality,” or “poor quality.”

Each study was assessed for potential bias based on the different components specified in the tool. The domains assessed for each study will be presented in the results section, into the corresponding tables. Two independent researchers assessed the risk of bias (G.L. and A.A.) and disagreements were resolved by discussion with a third author (F.C.).

## Results

A total of 365,857 studies were initially identified using the above procedure, including n.511 records from PubMed, n. 351,213 records from Web of Science and n. 14,133 records from Science Direct.

Records removed before screening were n. 93,478 failed to meet study design criteria, n. 270,943 failed to meet intervention criteria, n.132 due to the presence of study population with a diagnosis other than ASD and n. 57 duplicate records; after screening, the authors reviewed 1247 articles in more detail.

**Figure 1.**
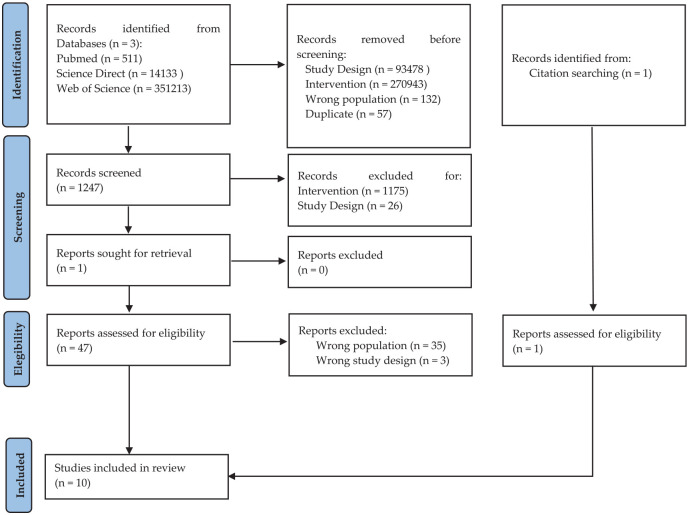
Prisma flow diagram for literature search strategy. From: Page et al. (2021). The figure outlines the search and review process with the total number of articles included and excluded in this review.

Applying our inclusion/exclusion criteria, n. 1175 were excluded for wrong intervention type, n. 26 were excluded for wrong study design. Of the n. 47 analyzed for eligibility, n. 35 were excluded for the wrong population involved and n. 3 for wrong study design. All details were reported in the PRISMA flowchart (Figure 1). The studies selected for the systematic review were conducted between 2005 and 2024 in North America (*n* = 3), Europe (*n* = 5), and Asia (*n* = 2). Of these, 4 studies (40%) used the Snoezelen multisensory room, 5 studies (50%) used the MSE, and only one study (10%) used the Snoezelen multisensory room, living room and stimulus preference rooms.

### Sample characteristics

All participants had a clinical diagnosis of ASD; of the included studies, n. 3 studies (30%) included a sample with ASD with moderate or profound ID. Only one study (10%) included ASD with the specification of language impairment. The remaining six (60%) included a sample of autistic individuals without further specification. Analyzing the 10 studies, the male/female ratio was reported in 8 studies. Among these, there was a total of 126 males and 45 females, with one study including only 3 males. Two studies did not provide information on gender distribution. The overall proportion of gender representation across the eight studies with available data was M:F = 2.8:1. The age range of participants in the reviewed studies was 3–52 years. Specifically, 5 studies (50%) included a sample of children, 4 studies (40%) included a sample of adults, and only 1 study (10%) included a sample of both children and adults. Among the studies included in this review, 9 (90%) explicitly utilized diagnostic tests to assess the participants. These tests included widely recognized tools such as the Childhood Autism Rating Scale (CARS), the Autism Diagnostic Interview (ADI), the Autism Diagnostic Observation Schedule (ADOS-2), and the Sensory Profile Questionnaire, among others. When it comes to the severity of autism, only two studies (20%) provided detailed information about the levels of severity within their sample. Specifically, these studies reported the severity using categories such as moderate to severe (levels 2–3), reflecting the participants’ functional abilities and the degree of support required. In terms of intellectual functioning, five studies (50%) reported specific information on IQ levels, ranging from mild to profound intellectual disability (ID; See Table 1).

**Table 1.** Summary of participant characteristics.

Reference	Diagnostic features	Sample size (EG; CG)	Age range (M; SD) in years	Number M: F	Ratio M: F	Diagnostic and clinical assessment	Severity of autism	Reported I.Q.
Basadonne et al., 2021	ASD	13	3–8 (5.57 ± 1.11)	10:3	3.3:1	ADOS-2	N.S	n.9 subjects > 70, n. 4 subjects < 70
		8	16–36 (24.8 ± 7.01)	5:3	1.66:1			n. 8 subjects < 70
De Domenico et al., 2024	ASD	10	3–6	9:1	9:1	PEP- 3, Cars 2	N.S	N.S
		10	3–6	8:2	4:1			
Fava & Strauss, 2010	(a) ASD with poor linguistic abilities	9	30–48 (37.8; N.A)	N.S	N.A	N.S	N.S	Profound ID
	(b) profound ID	9					N.A	Profound ID
	(c) profound ID, poor motor and linguistic abilities	9					N.A	Profound ID
Kaplan et al., 2006	ASD with moderate-profound ID	3	31–52 (43.3; N.A)	2:1	2:1	N.S	N.S.	n. 1 subject with moderate ID, n. 2 subjects with profound ID
McKee et al., 2007	ASD with moderate ID	3	28–32 (30.3; N.A)	3M	N.A	N.S.	N.S	Moderate ID
Mey et al., 2015	ASD	6	5–8 (6.3; N.A)	N.S	N.A	Sensory Profile Questionnarie	liv. 2–3 (moderate-severe)	N.S.
Min-Kyoung & Nam-Kyu, 2022	ASD	3	3–12 (6.3; N.A)	2:1	2:1	Short Sensory Profile	N.S	N.S.
Novakovic et al., 2019	ASD with moderate-severe ID	20	16–35 (23.70 ± 4.15)	29:11	2.33:1	CARS, ADI	liv. 2–3 (moderate-severe)	n. 2 subjects with mild ID, n. 9 subjects with moderate ID, n. 20 with severe ID, n. 9 with profound ID
		20						
Sawicki et al., 2024	ASD	20	6–17 (13.55 ± 3.41)	14:6	2.33:1	mYPAS, Watcha scale, FPS-R, PHBQ,	N.S.	N.S.
		20	6–17 (13.35 ± 2.43)	11:9	1.22:1			
Unwin et al., 2022	ASD	41	4–12 (8 ± 2.05)	33:8	4.12:1	ADOS 2, WASI-II, WPPSI-IV, RBS-R, SP, ASC-ASD;	N.S.	⩾70

ASD: autism spectrum disorder; EG: experimental group; CG: control group; M: mean; SD: standard deviation; M:F = Male:Famale; IQ: intelligence quotient; I.D: intellectual disability; NA: not applicable; ADOS-2: autism diagnostic observation schedule – second edition; PEP-3: psychoeducational profile—third edition; CARS: Childhood Autism Rating Scale; NS: not specificable; ADI: autism diagnostic interview – revised; mYPAS: Modified Yale Preoperative Anxiety Scale; FPS-R: Faces Pain Scale—Revised; PHBQ: Post Hospitalization Behavior Questionnaire; WASI-II: Wechsler Abbreviated Scale of Intelligence – Second Edition; WPPSI-IV: Wechsler Preschool and Primary Scale of Intelligence – Four Edition; RBS-R: Repetitive Behaviors Scale–Revised; ASC-ASD: Anxiety Scale for Children–ASD.

### Intervention type

The studies analyzed represent a broad typology of heterogeneous interventions. These interventions have different aims and use a wide variety of methods.

In some studies (n. 2, 20%; McKee et al., 2007; Novakovic et al., 2019), the intervention was carried out by experts in Snoezelen administration according to the standardized method (Fava & Strauss, 2010). In two studies, the intervention was carried out by occupational therapists (Kaplan et al., 2006; Min-Kyoung & Nam-Kyu, 2022), while in one study the caregiver was mainly involved (Fava & Strauss, 2010). The other five studies, in particular, programmed a specific intervention delivered by a therapist who was not further specified (De Domenico et al., 2024; Mey et al., 2015; Sawicki et al., 2024; Unwin et al., 2022).

Interestingly, 50% of the interventions took place in a daycare or residential center (Basadonne et al., 2021; Fava & Strauss, 2010; Kaplan et al., 2006; McKee et al., 2007; Novakovic et al., 2019); De Domenico et al. (2024) and Unwin et al. (2022) have been conducted in academic settings, one study was conducted in a preoperative dental clinical center (Sawicki et al., 2024). In the remaining studies, the setting was either not explicitly defined or not well specified, making it difficult to ascertain the context in which the interventions were conducted (Mey et al., 2015; Min-Kyoung & Nam-Kyu, 2022). The main effects investigated were on (1) prosocial behavior (n. 5, 50%); (2) repetitive or stereotyped behavior (n. 4, 40%), (3) challenging behavior interpreted as aggressive and destructive actions (n. 3, 30%), (4) attention and cognitive functions (n. 2, 20%), (5) sensory seeking behavior, including responsiveness or unusual interest to sensory input (n. 3, 30%). Two studies assessed the effect on anxiety behavior and positive affect. Specifically, one of these focused on anxiety levels prior to dental treatment. No adverse effects were reported. A summary of study characteristics and the main results is been provided below (see Table 2).

**Table 2.** Overview of study design, intervention, and outcome measures.

Reference	Study design	Aims	Outcome measures	Total n. of experimental sessions	Duration in weeks (frequency of sessions/week)	Session time (min)	Intervention experimental type	Setting	Intervention provider
Basadonne et al., 2021	Observational Study	To examine the effects on cognitive function	Observation forms on a 5-point Likert-type scale of sustained attention; selective attention; association; single inhibition; receptive communication; verbalization; turn.	5	1(5) or 5(1)	30	Multisensory interactive rooms; each session was attended by 1 participant at a time.	MSE	Therapist, not further specified
De Domenico et al., 2024	Rct Pilot Study	To explore the effectiveness of behavioral intervention in MSE on abnormal sensory responses and adaptive behaviors	PEP-3 and CARS-2	36	18(2)	45	MSE combined with standard treatment (psychomotor therapy)	MSE	Specialized therapist providing neuropsuchomotor rehabilitaion
Fava & Strauss, 2010	Observational Study	To compare effects on the (1) disruptive behavior and (2) pro-social behaviors of the Snoezelen environment and the Stimulus Preference environment	Coding scheme evaluated by blind occupational therapists and psychologists for frequency of (1) disruptive (including stereotypies) and (2) prosocial behaviors	20	7(3)	25	(A) Snoezelen approach, 1 participant per session with 1 caregiver maintaining non-directed contact, guiding the participant to appropriately use and activate equipment (B) Stimulus Preference, 1 participant per session with 1 caregiver maintaining non-directed contact, stimuli adapted to client’s preferences, guiding appropriate equipment use. (C) Living room, 5 participants with 3 caregivers, regularly scheduled activity times, group interaction with equipment during structured activities.	(A) Snoezelen room; (B) Stimulus preference;(C) Living room	Caregiver
Kaplan et al., 2006	Observational Study- ABA Design	To examine the effect on (1) pro-social and (2) challenging behaviors	Coding scheme for frequency of (1) spontaneous initiative and (2) challenging behaviors.	Unclear	N.S(2)	30	Occupational therapy, each session was attended by 1 participant at a time.	Snoezelen room	Occupational therapist
McKee et al., 2007	Case Series ABAB Reversal design	To evaluate the effects on (1) Disruptive and (2) Prosocial behaviors	Coding scheme for the number/session of targeted behaviors	56	4(Every day)	45	Snoezelen intervention, each session was attended by 1 participant at a time.	Snoezelen room	Nurses (certified in the Snoezelen administration)
Mey et al., 2015	Observational Study	To investigate the effects on sensory responses	6-point Likert scale on different sensory responses (auditory, visual, and tactile) filled by the parent	24	24(1)	60	The intervention was divided into 3 programs each dedicated to one sensory channel; each session was attended by 1 participant at a time with one caregiver	Snoezelen room	N.S
Min-Kyoung & Nam-Kyu, 2022	Case Series	To investigate the effects on personalized target behaviors of stimulating vs relaxing use of MSE	Frequency and duration of target repetitive behaviors of each participant	18	6(3)	30	The intervention focused on two sensory stimuli (visual and auditory) used in two different conditions: Stimulating versus relaxing MSE. Each session was attended by 1 participant at a time.	MSE	Occupational therapists
Novakovic et al., 2019	Rct	To investigate the effects on (1) ASD severity; (2) intensity of stereotyped or repetitive behaviors	(1) CARS total score; (2) CARS specific items: adaptation to change, object relationship, activity level.	36	12(3)	30	The intervention based on a non-directive and non-threatening approach; each session attended by groups of 3 participants at a time + usual daily scheduled activities in daycare centers	Snoezelen room	Special educator (expert in the Snoezelen administration)
Sawicki et al., 2024	Rct	To evaluate the impact of Multisensory room use on preoperative anxiety and postoperative outcomes in children with ASD undergoing dental treatment.	Modified Yale Preoperative Anxiety Scale; Puls oximeter; Watcha scale; Faces Pain Scale-Revised; Post Hospitalization Behavior Questionnaire for Ambulatory Surgery.	1	N.A	20	Multisensory room used during preoperative care	MSE	N.S.
Unwin et al., 2022	Observational Study	To investigate the effect of control over the sensory changes on (1) repetitive motor behaviors; (2) sensory behaviors; (3) social communication; (4) anxiety; (5) positive affect; (6) attention	Coding scheme for frequency and duration of (1) Repetitive Motor Behaviors; (2) sensory behaviors; (3) social communication; (4) anxiety; (5) positive affect; (6) attention	1	N.A	N.S	(A) Active-Change condition: the participants may change sensory aspects of the equipment themselves (B) Passive-Change condition, each respective piece of equipment, changes color every 3 s without the participant’s input.	MSE	Experimentalist

ASD: autism spectrum disorder; CARS: Childhood Autism Rating Scale; MSE: multisensory environment; N.A: Not applicable; N.S: Not Specificable; PEP-3: Psychoeducational Profile—Third Edition; RCT: randomized controlled trial.

### Outcome measures

Most studies used a coding scheme for the frequency and duration of targeted behaviors (n.5, 50%); of these, two (20%) also used Likert-type scales to document change. Three studies (30%) used standardized and validated outcome measures (De Domenico et al., 2024; Novakovic et al., 2019; Sawicki et al., 2024; See Table 2).

### Quality assessment

A total of 10 studies, published between 2005 and 2024, were included in this systematic review that investigated the effectiveness of using the multisensory room to treat the specific symptomatic features of autistic people. The characteristics of the included studies are shown in Tables 3 and 4. The sample includes seven non-randomized studies and three RCTs. Looking at the quality of the available literature according to the AHRQ standard, we found that 3 RCTs included in this review achieved ‘fair quality’; according to the NOS for non-randomized studies, we found that one study was of “poor quality,” 4 were of “fair quality” and 2 were of “good quality.” In all the RCTs included the random sequence generation was reported, instead of the allocation concealment method, which was not described in Novakovic’s and Antosh’s studies. Regarding sample size calculation, the method of power analysis was quite clear in all the studies included. Only 2 RCTs were single-blinded (De Domenico et al., 2024; Novakovic et al., 2019). In addition, except for Sawicki’s study, the risk of attrition bias for the included RCTs was low; it was clearly stated how many patients were screened, how many were excluded from randomization and why, and how many were lost to follow-up without specifying the reason. Flow diagrams showing the patient selection process were reported in all the included RCTs, apart from Novakovic’s and Sawicki’s studies. Finally, we found that only De Domenico’s protocol trial was registered in a public registry, which should be mandatory according to the Consolidated Standards of Reporting Trials (CONSORT) 2010 guidelines.

All seven included non-randomized studies were observational studies. The representativeness of the exposed cohort was well- defined in seven of these studies, and an adequate description of the exposure assessment based on objective data and structured interviews was fully reported in all seven studies. Regarding the selection of unexposed cohorts, only five studies clarified the exclusion criteria applied to the sample population. In addition, the assessment of outcomes that were not available at baseline was fully reported in all seven studies. Furthermore, three studies provided adequate follow-up, with an average of 3 months. Finally, we found that the comparability of the cohorts, based on the design or analysis and adjusted for important prognostic factors, was adequate in only two of the seven trials. The non-randomized studies included in the review had an overall moderate risk of bias. Table 4 shows the information on the risk domains for each study. There was good agreement between the two reviewers who performed this quality scale (G.L. and A.A.). The characteristics of the included studies are shown in Tables 3 and 4.

**Table 3.** Assessing study quality: Newcastle–Ottawa scale analysis results.

Publication	Exposed representation	Ascertainment of exposure	Selection of the non-exposed	The outcome was not present at the start of the study	Comparability of cohorts	Assessment of outcome	Sufficient follow-up time	Adequacy of follow-up of cohorts	Total	Overall quality
Basadonne et al., 2021	1	1	1	1	0	1	0	0	5	Fair
Fava & Strauss, 2010	1	1	1	1	2	1	1	1	9	Good
Kaplan et al., 2006	1	1	0	1	0	1	1	0	5	Fair
Mckee et al., 2007	1	1	1	1	0	1	0	0	5	Fair
Mey et al., 2015	1	1	1	1	2	1	1	1	9	Good
Min-Kyoung & Nam-Kyu, 2022	1	1	1	1	0	1	0	0	5	Fair
Unwin et al., 2022	1	1	0	0	0	1	0	0	3	Poor

**Table 4.** Quality assessment of the included study by using the Cochrane Risk of Bias Tool for RCT and the AHRQ standards. *“Good quality”: All criteria met* (i.e. low for each domain); “Fair quality”: One criterion not met (i.e. high risk of bias for one domain) or 2 criteria, and the assessment that this was unlikely to have biased the outcome, and there is no known important limitation that could invalidate the results; “Poor quality”: One criterion not met (i.e. high risk of bias for one domain) or 2 criteria unclear, and the assessment that this was likely to have biased the outcome, and there are important limitations that could invalidate the results; Poor quality: two or more criteria listed as high or unclear risk of bias.

Publication	Random sequence generation	Allocation concealment	Selective reporting	Other bias	Blinding of participants and personnel	Blinding of outcome assessment	Incomplete outcome data	AHRQ standard
De Domenico et al., 2024	Low	Low	Unclear	Unclear	Low	Low	Low	Fair
Novakovic et al., 2019	Low	Unclear	Unclear	Unclear	Low	Low	Low	Fair
Sawicki et al., 2024	Low	Low	Unclear	Unclear	Low	Low	Low	Fair

### Synthesis of main effects

#### Prosocial behaviors

Prosocial behaviors were defined as all voluntary social interaction actions designed to engage, show, or help another person. Five studies focused on these core autistic symptoms.

In the study by Unwin et al. (2022), the authors focused on analysis following treatment in an MSE on social communication (this consisted of five measures: social behavior, gestures, mimicry, speech, and rapport). The frequency and duration of these behaviors were coded separately for analysis. The measure of social behavior included the analysis of several items: showing, requesting, offering information, asking for information, and sharing pleasure; while the measure of gestures included conventional, informational, emphatic, and deictic. For the speech measure (speech, stereotyped/idiosyncratic speech, and vocalizations) both frequency and duration were coded.

The authors did not find any improvement in pro-social behavior: no significant differences were found between conditions for speech and for the frequency of the other variables (social behavior, gestures, mimicry, anxiety, and positive affect behavior).

Fava and Strauss (2010) examined both disruptive and pro-social behaviors in adults with profound mental retardation and autism. Pro-social behaviors in this context included active behaviors toward sensory stimuli (such as glances, smiles, and purposeful body and arm movements) and social behaviors toward caregivers (such as approaching behaviors and explicit requests for social contact). While the frequency of pro-social behaviors did not increase after treatment in the Snoezelen room, a statistically significant improvement in pro-social behaviors was observed in participants exposed to the Stimulus Preference condition, where the environment was adapted to individual preferences.

Basadonne et al. (2021) evaluated different target behaviors: (1) receptive communication (RC), interpreted as the ability to understand instructions; (2) verbalization (V), or the ability to produce words or sentences at the request of the therapist; (3) turn (T), the ability to consider the presence of other subjects and to recognize and respect their role in the interaction. After the intervention, the authors found a significant improvement in RC, V, and T only in the sample of children.

Finally, in the case series by Kaplan et al. (2006), they report a positive trend toward more prosocial behavior, defined as the number of times the patients initiated an activity or remained engaged with staff, after the MSE intervention, but without a significant result. Similarly, the three patients observed by McKee et al. (2007) showed a trend toward more prosocial behavior, but without statistical significance.

#### Repetitive and stereotyped behaviors

A total of n. 4 studies investigated the usefulness of MSE intervention for repetitive and stereotyped behavior. Only one study included children (Unwin et al., 2022); the other studies included adolescents and young adults. The outcome measures used, although mostly standardized, were mainly based on operator observation. Only Fava and Strauss (2010) and Unwin et al. (2022) assessed the frequency of stereotyped behaviors through videotaped sessions.

Novakovic et al. (2019) showed that continuous sessions in the Snoezelen^®^ room had positive effects on subjects with ASD accompanied by low intellectual functioning. The sample was divided into a control group and an experimental group; assessments were made using the CARS before and after 3 months of treatment. The authors found that the intervention in a Snoezelen environment had positive effects, with a significant decrease in ASD severity. In addition, a statistically significant difference was found in the experimental group in three specific areas of the CARS: adaptation to change, object relationship, and activity level. These three items were interpreted by the authors as being inherent to repetitive or stereotyped behaviors.

Unwin et al. (2022) considered repetitive behavior, measured as repetitive whole-body movements, repetitive hand/finger/foot movements, and repetitive locomotor movements, which were combined to produce an overall score. The interventions studied consisted of two different conditions: (1) the active change condition, where participants had control over the MSE device; (2) the passive change condition, where the MSE device changed automatically without any input from the participant. The study showed a statistically significant reduction in the frequency and duration of repetitive behaviors and stereotyped/idiosyncratic vocalizations in the active-change condition compared to the passive-change condition.

Fava and Strauss (2010) investigated whether Snoezelen and Stimulus Preference environments have different effects on some behaviors in adults with severe intellectual disability and autism compared to a normal living room. The authors found that the frequency of stereotyped behaviors decreased significantly after treatment for both autistic subjects who received the Snoezelen intervention compared to the other conditions.

Min-Kyoung and Nam-Kyu (2022) investigated the effects of an MSE on restricted target behaviors, including looking sideways, sucking fingers, flapping hands, stereotyped vocalizations, and oral exploration. The authors selected three autistic subjects and then modulated the visual and auditory MSE stimuli according to each participant’s sensory profile, creating two main settings: a relaxing condition and a stimulating condition. The results showed that MSE could influence the participants’ repetitive behaviors. In particular, the stimulating MSE settings, characterized by the integration of visual and auditory stimuli, were the most effective intervention, demonstrating that the integration of visual and auditory stimuli increases positive changes compared to the single intervention.

#### Disruptive and aggressive behaviors

A total of three studies investigated the effect of MSE intervention on disruptive and aggressive behavior. Both studies included autistic individuals with intellectual disability aged 28–52 years.

The three patients observed by McKee et al. (2007) did not show a reduction in aggressive and disruptive behaviors.

Fava and Strauss (2010), who assessed disruptive behaviors through videotaped sessions, showed a significant reduction in frequency in autistic people who attended Snoezelen.

In the case series by Kaplan et al. (2006), measures of challenging behaviors were examined on the days following the MSE therapy session. The authors showed a small but noticeable reduction in frequency during the day after the Snoezelen intervention.

#### Cognitive functions

Only two authors have assessed efficacy on cognitive functioning, mainly attention.

Basadonne et al. (2021) investigated whether interactive activities carried out in an MSE can improve the effectiveness of therapeutic intervention in a sample of children and young autistic adults. In particular, the authors hypothesized that the ability to calibrate sensory stimulation offered by the MSE could modulate positive emotional states and improve the yield of the intervention on cognitive function. Specifically, the authors measured improvements using 5-point Likert-type scale observation forms, with scores reflecting the degree of assistance needed to complete a task related to a targeted function. The authors found significant improvements in sustained and selective attention, and finally in inhibition, or the ability to refrain from doing something. Conversely, no statistically significant improvement in performance was found in the adolescent/adult group.

Unwin et al. (2022) assessed the attention of autistic subjects in the sensory room. Specifically, the authors measured attention as the ability to maintain focus on a task. The authors found increased attention when participants had control over the MSE equipment (the active change condition).

#### Sensory behaviors

In the field of peculiar sensory reactivity, only three studies investigated variables inherent to hyper- or hypo-responsiveness to sensory inputs or unusual interest in sensory aspects of the environment.

Mey et al. (2015) conducted a study to test the effects of a specific intervention program in MSE in autistic children. The study focused on three main sensory areas: visual, auditory, and tactile, measured by a 6-point Likert-type scale completed by parents. A qualitative analysis of the results showed better adaptation to sensory stimuli, but no statistical results were found.

A similar result was found by Unwin et al. (2022). The authors used measures of specific behaviors classified as sensory seeking or sensory defending in the auditory, visual, and tactile domains. Three outcome measures were created: sensory seeking behaviors, sensory defending behaviors, and total sensory behaviors measured in duration and frequency. The study found that sensory-seeking behaviors were significantly fewer and shorter when the participant had control over sensory stimuli than in the passive change condition. In contrast, there were very few sensory defensive behaviors in both conditions and no difference in frequency or duration.

De Domenico et al. (2024) also explored sensory behaviors in autistic children through the use of an MSE combined with psychomotor therapy. Their study found that the intervention group showed significant improvements in adaptive responses to sensory stimuli across multiple domains, particularly in reducing sensory defensiveness and increasing appropriate sensory-seeking behaviors. Parents reported noticeable changes in their children’s ability to regulate their responses to taste, smell, and touch. These findings further supported the potential role of MSEs in modulating sensory behaviors in autistic children, promoting better adaptation to sensory inputs in daily activities.

#### Other therapeutic target

In the study by Unwin et al. (2022), in addition to the assessment of repetitive and pro-social behaviors already discussed in the previous paragraph, the authors focused on analysis following treatment in an MSE for anxiety (whining/whimpering, stuttering, trembling/shaking body or voice, jumping, body contortions/rigidity, physical discomfort, desire to leave, verbal expression of fear or worry, crying and irritability). The frequency of each behavior was recorded, and the variables were summed to give an overall anxiety score. The authors found no difference in anxious feelings/behaviors between the conditions. In addition, the authors also looked at positive affect, with frequency and duration of smiling, laughing, and verbal expressions of pleasure. No significant effect was reported for positive affect behaviors in either condition.

Sawicki et al. (2024) further explored the use of a multisensory room in autistic pediatric patients to reduce anxiety and fears before operative dental treatment. Their RCT found a significant reduction in preoperative anxiety levels for children placed in the MSE compared to those in standard preoperative environments. Using both behavioral observations and parental reports, the study demonstrated that the MSE provided a calming effect, particularly in the critical moments before surgery, leading to fewer observable anxiety behaviors (such as trembling, crying, and verbal expressions of fear).

## Discussion

The last 15 years have seen an extraordinary expansion in the use of MSE in educational, therapeutic, and recreational settings for autistic children and adults, with a rapid proliferation of such environments even in community care centers and schools (Lotan & Gold, 2009). However, data in the literature were limited, both in terms of number of studies and sample size, so this finding should be interpreted with caution.

To our knowledge, this is the first systematic review to assess the effectiveness of a wide range of interventions in MSE for autistic children and adults. Although there were no time or language restrictions, our search identified only 10 8 studies out of 1247 screened articles, of which 3 had a very limited sample size (n. 3 participants). Overall, this systematic review found some evidence for the efficacy of MSE as an intervention for ASD in a small number of studies, and the sample appears evenly distributed in terms of the number of studies for both adults and children. However, the total numbers appear to be very limited, with a sample mean of 12.75 (*SD* 9.72, sample range 3–41) patients per study arm. The types of intervention often differ between studies in terms of aims, intervention provider, frequency of measures or instruments used, and total number of sessions (Table 4). Conversely, the setting is consistent. Indeed, in all the included studies in our review, MSE is intended as a separate context from everyday environments, minimizing their ecological validity, as also reported in studies in which SAEs with more complex technology (Williams et al., 2024).

Improvement of social functioning appears to be the primary objective of most of the included studies. However, only one study showed that intervention in a standardized MSE can promote prosocial behaviors (Basadonne et al., 2021). It is interesting to note that the authors showed an improvement in the pediatric population but not in the adolescent and young adult groups. This finding may be due to the choice of outcome measures. The behaviors assessed (RC, VI, and T) seem to be more appropriate therapeutic targets for the pediatric age group than for adolescents and young adults. In adulthood, levels of expressive and receptive language appear to be stable over time. Moreover, selected behaviors such as receptive communication and verbalization may be particularly influenced by the verbal skills attained by the subjects. The body of knowledge supported the extreme variability of verbal ability in autism (La Valle et al., 2024); specifically, individuals who have co-occurring intellectual disability generally presented delays and/or limited verbal skills that tend to have a stable trajectory across time (Pickles et al., 2020; Solomon et al., 2023; Szatmari et al., 2015; Waizbard-Bartov et al., 2022). In the sample of this study, children had a higher IQ mean than adults, a difference that might have influenced the effectiveness.

Another interesting result emerged from Fava and Strauss (2010). Indeed, the authors compared an MSE conformed to the Snoezelen method and a customized MSE (Stimulus Preference room) based on the individual preferences of each participant. The author noticed an increase in social behaviors toward caregivers only in an MSE adapted to individual favorite stimuli, and pointed to personal preferences as potential influencing factors. A similar result would seem to emerge from another of the included studies. Unwin et al. (2022) verified the presence of greater improvements when participants had direct control over the MSE equipment, which appears to reduce sensory and psychological distress, improve their ability to modulate external stimuli and create a favorable atmosphere, or reinforce through sensory pleasant consequences (Piazza et al., 2000; Rapp, 2006).

In the area of unusual interest in sensory aspects of the environment, Mey et al. (2015), Unwin et al. (2022), and De Domenico et al. (2024) examined the effects of MSE interventions. However, the preliminary description of baseline sensory profiles was unclear. The authors showed that sensory-seeking behaviors were significantly less and shorter when the participant had control over sensory stimuli. The effect of MSE intervention on sensory reactivity may be the most relevant. Furthermore, Unwin et al. (2022) and De Domenico et al. (2024) highlight that sensory-seeking behaviors were significantly fewer and shorter in MSE under their control.

As discussed above, this result might have broad potential in terms of personalized therapeutic settings tailored to individual needs. In addition, it could be an interesting strategy to help autistic individuals learn to manage sensory difficulties encountered in daily life through MSE settings, one of those needs and priorities directly expressed by the autistic community (Pellicano et al., 2014). Indeed, several sensory processing patterns characterized autistic children, with early sensory sensitivity playing an important role in the later development and diagnosis of social interactions and restricted/repetitive behaviors (Kojovic et al., 2019; Riboldi et al., 2024; Schulz & Stevenson, 2019). Based on the now clear assumption that each child has a specific sensory profile (Riboldi et al., 2024), further research is needed to explore the wider implications of equipment preferences and sensory responses within MSE settings.

Several studies have investigated the usefulness of MSE in reducing repetitive and stereotyped behaviors, frequently observed in autistic individuals. MSEs would seem to have a positive effect in decreasing these behaviors, as evidenced by four studies (Fava & Strauss, 2010; Min-Kyoung & Nam-Kyu, 2022; Novakovic et al., 2019; Unwin et al., 2022). More recent theories have suggested that stimming may tend to increase under conditions of over-arousal, stress, anxiety, boredom, or sensory deprivation (Joyce et al., 2017; Leekam et al., 2011; Mahone et al., 2004; Muthugovindan & Singer, 2009). Conversely, the frequency or duration can decrease when the individual engages in pleasurable or relaxing activities, suggesting that sensory regulation plays a crucial role in behavior management (Baillon et al., 2002). Most of these studies assessed the frequency and duration of these behaviors during or immediately after the session in MSE, emphasizing the possible role-played by sensory stimulation in reducing states of emotional tension.

However, more attention should be paid in future studies to consider these non-harmful forms of behaviors as outcome measures of the core symptoms of autism. Indeed, stereotyped behaviors can have different personal significance, such as being used as a mechanism for self-regulate a state of emotional hyperarousal (Pellicano & Burr, 2012) or to promote attention or amusement. Therefore, the present findings may be useful if interpreted as a means to foster more personalized and less oppressive therapeutic environments for autistic people (Kapp et al., 2019; Williams et al., 2024).

Challenging behaviors were another aim evaluated by three studies, with mixed results (Fava & Strauss, 2010; Kaplan et al., 2006; McKee et al., 2007). Specifically, these three reports were all on adult samples but differ strongly in frequency of intervention; in addition, they should be interpreted with caution due to small sample sizes. Mixed results were reported also about perceived levels of anxiety. While Unwin et al. (2022) did not note any reduction in perceived levels of anxiety, measured through behaviors defined by the authors in a baseline condition. The recent study conducted by Sawicki et al. (2024) explored the preoperative use of MSE in autistic pediatric individuals, in an RCT. This study showed that waiting in an MSE significantly reduced anxiety during the preoperative phase, highlighting the potential of MSE in reducing stress and anxiety in particularly stressful clinical settings, such as preparation for surgery. This was the only included study evaluating the effectiveness of MSE in such a specific condition. Nonetheless, it can be an interesting and useful way to use MSE to foster autonomy and self-management skills in real-life situations experienced as stressful or dangerous (Pellicano et al., 2014).

Finally, interventions to control external sensory stimuli in everyday environments have shown benefits for autistic individuals (Ikuta et al., 2016; Kinnealey et al., 2012; Pfeiffer et al., 2019; Rowe et al., 2011; Williams et al., 2024). Broadly, these adaptations have led to improvements in attention and learning (Kinnealey et al., 2012; Pfeiffer et al., 2019; Rowe et al., 2011). Indeed, the use of MSE may provide a controlled environment that can calibrate the frequency, intensity, and duration of sensory stimuli, which may lead to a reduction in defensive sensory behaviors (Ashburner et al., 2013; Robertson & David, 2015; Unwin et al., 2022). Furthermore, multisensory stimulation based on the characteristics and needs of the patient may favor exploratory behaviors and implicit learning by providing tasks with immediate sensorimotor feedback (Settimo et al., 2023). Unfortunately, only two studies have investigated this facilitative aspect of MSE (Basadonne et al., 2021; Unwin et al., 2022), reporting an improvement in the ability to stay focused on a task and in sustained and selective attention in children.

Collectively, these findings highlight the important role of environmental factors in modulating attentional processes and maladaptive behaviors in ASD (Williams et al., 2024). More importantly, they also highlight the potential of MSE: tailoring the environment to the sensory needs of the individual could provide a stimulating and controlled setting in which to learn more comfortably.

## Limitations and future directions

Our results should be considered with several limitations. The analysis showed that some of the included studies had small sample sizes, which may have underestimated the effects and made them underpowered. In addition, most of the studies were not RCTs and some did not include control groups. Another limitation was the heterogeneity of the studies in terms of target behavior, assessment measures, and intervention strategy. These differences made it difficult to organize a synthesis of the results. A further source of heterogeneity was the variability in the frequency and duration of the MSE interventions across the included studies. In some cases, interventions were administered two or three times per week, while in other instances, were not even reported. This variability may have influenced the outcomes, introducing a confounding factor and complicating direct comparisons between studies. More robust studies with standardized intervention for frequencies and durations, and larger sample sizes are needed to improve the consistency and interpretability of results. It appears that none of the studies explicitly monitored or reported adverse outcomes such as negative side effects or risks during the interventions. This represents a potential gap in the research design, especially since monitoring for adverse outcomes is crucial in autism intervention research to ensure the safety and well-being of participants. Although this review is not a meta-analysis, to our knowledge it is the first systematic review of MSE interventions in ASD. It collected all the data in the literature to date and may lead to a clearer and more objective assessment of the scientific evidence produced. One key consideration in this review was the inclusion of both child and adult samples, which was done to capture the full range of MSE interventions across different developmental stages. Including both age groups offers a comprehensive overview of the usefulness of MSE, as ASD presents distinct challenges at various life stages, particularly in relation to sensory reactivity and behavioral responses. This wide age range allows for the exploration of potential similarities and differences in MSE effectiveness across different age groups, which could provide valuable insights into how MSE interventions can be tailored to specific age-related needs. However, this approach also comes with certain limitations. The developmental differences between children and adults, including variations in neural plasticity, cognitive abilities, and sensory profiles, may have influenced the outcomes observed in the studies. These differences could make it challenging to draw specific conclusions that apply to both age groups. Further studies that examine the effects of MSE interventions on well-defined, age-specific subgroups are necessary to refine our understanding of how these interventions can be most effective for different populations. Finally, the inclusion of both children and adults in this review strengthens the scope of the findings by highlighting the potential for MSE to be useful across the lifespan of autistic individuals, but it also underscores the need for age-specific guidelines to optimize interventions for each developmental stage. Another limitation was the lack of detailed characterization of the selected samples (e.g. level of ASD severity, intellectual disability, language ability, comorbidities, sensory profiles). Further research on well-defined samples is needed to explore which types of sensory stimulation are most beneficial for specific patients, to better tailor interventions, and to develop useful guidelines. Despite these limitations, this systematic review of the current literature can highlight the potential of MSE interventions and suggest methodological strategies to improve in future studies. In addition, it may serve as a useful resource for those unfamiliar with the topic, helping to explain the benefits of MSE for both children and adults with ASD.

## Conclusion

MSE appears to be a useful tool for intervention in ASD, particularly in reducing the frequency of stereotyped and challenging behaviors; it may also be valuable in modulating sensory responses and improving engagement. In addition, the results may provide valuable insights for the development of future studies on the usefulness of MSE in adapting and personalizing the therapeutic setting to the individual’s sensory needs. However, as mentioned above, there are still few studies, and sample sizes are often small and poorly characterized, so there is a need for more and larger studies on this topic in the future.

## Supplemental Material

sj-docx-1-aut-10.1177_13623613251320424 – Supplemental material for The use of multisensory environments in children and adults with autism spectrum disorder: A systematic reviewz Supplemental material, sj-docx-1-aut-10.1177_13623613251320424 for The use of multisensory environments in children and adults with autism spectrum disorder: A systematic reviewz by Simona Leonardi, Marcella Di Cara, Silvia Giliberto, Adriana Piccolo, Carmela De Domenico, Giulia Leonardi, Angelo Alito, Rosamaria Siracusano, Rocco Salvatore Calabrò, Angelo Quartarone and Francesca Cucinotta in Autism
