# Supplementary material for: The use of multisensory environments in children and adults with autism spectrum disorder: A systematic review
Source: Autism. 2025 Mar 14;29(8):1921–38. doi: 10.1177/13623613251320424 (PMC12255839; doi:10.1177/13623613251320424)
Supplement: sj-docx-1-aut-10.1177_13623613251320424 – Supplemental material for The use of multisensory environments in children and adults with autism spectrum disorder: A systematic reviewz [file sj-docx-1-aut-10.1177_13623613251320424.docx]

***Supplementary file - Supplemental material for “The use of multi-sensory environments in children and adults with autism spectrum disorder: a systematic review”***

Additional details on search strategy, for “The use of multi-sensory environments in children and adults with autism spectrum disorder: a systematic review” in Autism

Two seasoned information specialists (SG and SL) crafted the search strategy in accordance with the PRISMA guideline for reporting. Keywords were carefully curated based on expert opinions.

The following electronic databases and international trial registries were searched PubMed, Web of Science, and Science Direct.

Search syntax for each database (in alphabetical order)

1. PUBMED

The search strategy was defined by identifying four mash terms related to: ("sensorial"[All Fields] OR "sensorially"[All Fields] OR "sensory"[All Fields]) AND "room"[All Fields]) OR (("sensorial"[All Fields] OR "sensorially"[All Fields] OR "sensory"[All Fields]) AND ("environ"[All Fields] OR "environment"[MeSH Terms] OR "environment"[All Fields] OR "environments"[All Fields] OR "environment s"[All Fields] OR "environs"[All Fields])) OR (("multisensorial"[All Fields] OR "multisensory"[All Fields]) AND "room"[All Fields]) OR "snoezelen"[All Fields]) AND ("autism s"[All Fields] OR "autisms"[All Fields] OR "autistic disorder"[MeSH Terms] OR ("autistic"[All Fields] AND "disorder"[All Fields]) OR "autistic disorder"[All Fields] OR "autism"[All Fields] ).

1. SCIENCE DIRECT

The search strategy was defined by identifying four mash terms related to: ("autism" OR "autism spectrum disorders" OR "ASD") AND ("sensorial" OR "sensororially" OR "sensory") AND ("multisensory" AND “room” OR “snoezelen”).

1. WEB OF SCIENCE

The search strategy was defined by identifying four mash terms related to: TI= (Autism OR Autism spectrum disorder OR asd) AND TI=(sensorial OR sensory OR sensorially AND room) AND TI=(sensorial OR sensory OR sensorially AND envoirment) AND TI=(multisensorial OR multisensory AND room OR snoezelen);
